# The combined influence of earlier menopause and cardiac function on brain health

**DOI:** 10.1002/alz70855_100381

**Published:** 2025-12-23

**Authors:** Tallinn Splinter, Madeline Wood Alexander, Amy Kirkham, Gillian Einstein, Sandra E. Black, Jennifer S Rabin

**Affiliations:** ^1^ Sunnybrook Research Institute, Toronto, ON, Canada; ^2^ University of Toronto, Toronto, ON, Canada; ^3^ University of Toronto Scarborough, Toronto, ON, Canada

## Abstract

**Background:**

There are many menopauses, all marking a significant endocrine transition characterized by the depletion of ovarian hormones, including estradiol and progesterone. This transition, especially when it occurs earlier than average, is associated with increased risk for cardiovascular disease (CVD) and dementia. CVD contributes to cerebral small vessel disease and dementia, both partly via reduced cardiac output which can lead to cerebral hypoperfusion. Despite these established links, there is limited research examining the combined impact of age at menopause and cardiac function on brain and cognitive outcomes. This study investigated whether earlier age at menopause influences the associations between cardiac function and gray matter volume, white matter hyperintensity (WMH) burden, and cognitive performance.

**Method:**

We analyzed data from healthy postmenopausal female participants enrolled in both the Canadian Alliance for Healthy Hearts and Minds study and the Ontario Health Study. Cardiac function was assessed using resting left ventricular ejection fraction (LVEF) measured on cardiac MRI. Brain MRI was used to quantify total gray matter volume and WMH burden. Cognition was assessed with the Montreal Cognitive Assessment (MoCA) and the Digit Symbol Substitution Test (DSST). Linear regression models assessed the interactive associations of age at menopause and LVEF on brain and cognitive outcomes, adjusting for age, race/ethnicity, education, menopausal hormone therapy, cause of menopause (surgical/spontaneous), visceral adipose tissue, systolic blood pressure, and intracranial volume (for brain outcomes).

**Result:**

We included 708 participants (Mean age=63±5.4 years, range=45‐76; mean age of menopause=50±5.5 years, range=25‐61, mean LVEF=65±6.3%, range=45‐80%). Age at menopause moderated the association between LVEF and brain outcomes, such that earlier age at menopause strengthened the associations of lower LVEF with reduced gray matter volume (*p* = 0.03) and increased WMH burden (*p* = 0.008). The interactive associations between age at menopause and LVEF on cognitive outcomes were not significant (MoCA: *p* = 0.50, DSST: *p* = 0.26).

**Conclusion:**

Earlier menopause and reduced cardiac function may have a compounding negative effect on brain health. These findings underscore the importance of integrating sex‐specific factors, such as age at menopause, into investigations of risk factors for dementia. Further research is needed to elucidate the underlying mechanisms by which earlier menopause contributes to CVD‐related dementia risk.

